# Biochemical Analysis of the Role of Leucine-Rich Repeat Receptor-Like Kinases and the Carboxy-Terminus of Receptor Kinases in Regulating Kinase Activity in *Arabidopsis thaliana* and *Brassica oleracea*

**DOI:** 10.3390/molecules23010236

**Published:** 2018-01-22

**Authors:** Eun-Seok Oh, Yeon Lee, Won Byoung Chae, Jana Jeevan Rameneni, Yong-Soon Park, Yong Pyo Lim, Man-Ho Oh

**Affiliations:** 1 Department of Biological Sciences, College of Biological Sciences and Biotechnology, Chungnam National University, Daejeon 34134, Korea; oes0318@naver.com (E.-S.O.); yeonlee@cnu.ac.kr (Y.L.); yspark2005@gmail.com (Y.-S.P.); 2 Vegetable Research Division, National Institute of Horticultural and Herbal Science, RDA, Wanju 55365, Korea; chaeddang@korea.kr; 3 Department of Horticulture, College of Agriculture and Life Science, Chungnam National University, Daejeon 34134, Korea; saijeevan7@gmail.com (J.J.R.); yplim@cnu.ac.kr (Y.P.L.)

**Keywords:** *Arabidopsis thaliana*, brassinosteroid, *Brassica oleracea*, leucine-rich repeat receptor-like kinases, phosphorylation

## Abstract

Protein post-translational modification by phosphorylation is essential for the activity and stability of proteins in higher plants and underlies their responses to diverse stimuli. There are more than 300 leucine-rich repeat receptor-like kinases (LRR-RLKs), a major group of receptor-like kinases (RLKs) that plays an important role in growth, development, and biotic stress responses in higher plants. To analyze auto- and transphosphorylation patterns and kinase activities in vitro, 43 full-length complementary DNA (cDNA) sequences were cloned from genes encoding LRR-RLKs. Autophosphorylation activity was found in the cytoplasmic domains (CDs) of 18 LRR-RLKs; 13 of these LRR-RLKs with autophosphorylation activity showed transphosphorylation in *Escherichia coli*. BRI1-Associated Receptor Kinase (BAK1), which is critically involved in the brassinosteroid and plant innate immunity signal transduction pathways, showed strong auto- and transphosphorylation with multi-specific kinase activity within 2 h of induction of *Brassica oleraceae* BAK1-CD (BoBAK1-CD) in *E. coli*; moreover, the carboxy-terminus of LRR-RLKs regulated phosphorylation and kinase activity in *Arabidopsis thaliana* and vegetative crops.

## 1. Introduction

Plants encounter a variety of environmental stresses and thus must constantly balance their growth and defense responses. Plant growth and development are coordinated and regulated by diverse signaling pathways involving communication via inter- and intracellular signaling components. For instance, phytohormones are signaling messengers that help mediate the initiation and/or crosstalk of the various signaling pathways in higher plants, and they have attracted considerable recent interest, including into how their interactions with receptor proteins trigger rapid biochemical changes and induce intracellular gene expression and post-translational modifications [1].

Regarding this important research field, receptor kinases are upstream signaling components controlling many essential processes related to growth, development, and responses to the environment. *Arabidopsis thaliana* receptor-like kinases (RLKs) belong to a monophyletic gene family of 610 members that make up more than 2% of predicted protein-encoding genes in this plant. Results from *Arabidopsis* suggest that plant RLKs are involved in the tight control of plant growth and morphogenesis, resistance to diverse pathogens, and responses to biotic and abiotic stresses [2,3,4,5]; however, despite such studies, the functions of most plant RLKs remain unknown. The *Arabidopsis* RLKs can be divided into over 20 families based on the structure of the extracellular ligand-binding domain containing Leucine-Rich Repeat (LRR) sequences. The *Arabidopsis* LRR-RLK family can be further classified into 13 subfamilies based on sequence alignments of their cytoplasmic kinase domains (KDs). The best-characterized LRR-RLKs are Brassinosteroid Insensitive 1 (BRI1) and BRI1-associated receptor kinase 1 (BAK1); both are protein kinases critically involved in the brassinosteroid signaling pathway [6,7] and show multi-specific kinase activity [8]. Early studies found that BRI1 autophosphorylated serine and threonine residues exclusively in vitro; subsequently, numerous specific phosphorylated serine and phosphorylated threonine sites were identified [9]. Several sites of tyrosine autophosphorylation have also been identified, establishing that BRI1 is a multi-specific kinase [10].

Plant LRR-RLKs consist of an extracellular domain connected to the cytoplasmic domain (CD) of the protein by a single-pass transmembrane domain. The CD contains a juxtamembrane domain (JM), KD, and C-terminal (CT) polypeptide. Although the KD contains the structures and residues required for phosphoryl transfer from adenosine triphosphate (ATP) to amino residues or other protein substrates of downstream components, the C-terminus of receptor kinases often plays an important regulatory role and this function may be modulated by the phosphorylation of serine, threonine, and tyrosine residues within the CD. In the case of the brassinosteroid receptor, BRI1, the JM is an activator of the BRI1 KD [10] when the JM is truncated by 60 residues. Although studies of carboxy-terminus deletions have been reported from animal systems, little is known about the roles played by flanking domains, including the CT region, in other receptor kinases, and it remains unclear whether and how the flanking domains contribute to receptor kinase activity in *A. thaliana* and vegetative crops. In this study, we analyzed the auto- and transphosphorylation patterns and kinase activities of 43 *Brassica oleracea* LRR-RLKs (BoLRR-RLKs) and ten *A. thaliana* LRR-RLKs (AtLRR-RLKs) in vitro by comparing proteins with a full-length CD with those in which the flanking domain of the CT region was deleted. Interestingly, in the absence of the CT region, autophosphorylation kinase activity of AtLRR-RLKs was significantly inhibited in vitro. Overall, the results establish an important role for the CT region of LRR-RLKs in higher plants.

## 2. Results and Discussion

The mechanism of action of many animal receptor kinases is well characterized. It generally involves ligand-mediated homo- or heterodimerization of the receptor followed by auto- and transphosphorylation, and activation of the intracellular KD. Activation of receptor kinases results in protein recognition and interaction, leading to phosphorylation of downstream components of the signal transduction pathway and ultimately to changes in gene expression and cellular responses [6]. The large number of LRR-RLKs in higher plants, including *A. thaliana*, *Brassica rapa*, and *B. oleracea*, provides extensive opportunities for post-translational modification, including phosphorylation and protein-protein interactions, and thus enables the diversification and amplification of the signaling pathways regulating plant growth and development and responses to stresses, including many different pathogens [11].

In the comparative phylogeny analysis, we have used the peptide sequences of *A. thaliana, B. oleracea and Raphanus sativus* and constructed the neighbour joining (NJ) tree. The *LRR-RLK* genes in the phylogeny are grouped into 17 sub-groups (I-XVII) (Figure S1) based on the node support and the genes that have less node support are not considered under any sub-group (Figure S1). In addition, among the seventeen sub-groups most of the genes are found in sub groups II (21), XV (17) and XVI (14) and least number of genes are grouped in I, VII and XVII with three genes each. Moreover, in the phylogenetic tree genes of the three species are distributed unevenly among the sub-groups and they also have high bootstrap values indicating their phylogenetic relationship among the genes. In addition, as seen in the phylogeny (Figure S1), the *B. oleracea LRR-RLK* genes are mostly clustered with *R. sativus LRR-RLK* genes with high node support compared with model plant *A. thaliana* genes indicating that the genes of these two species may have high structural similarity and also these genes might have similar functional roles in respective plants.

**Figure 1:**
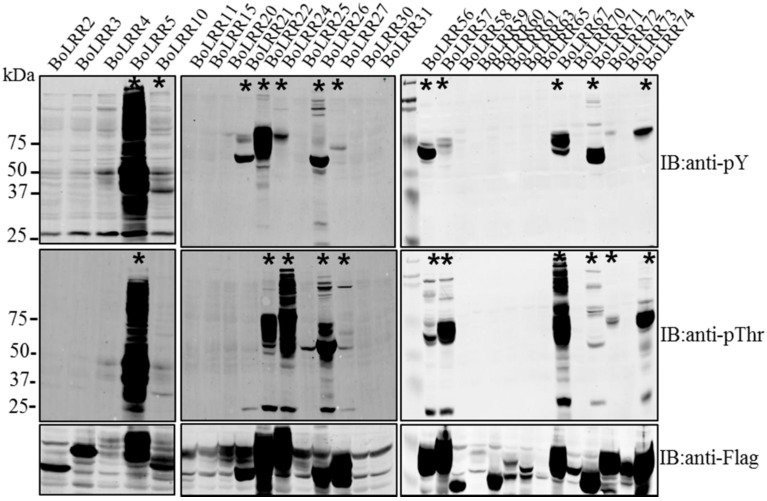
Auto- and transphosphorylation of recombinant *Brassica oleracea* LRR-RLK (BoLRR-RLK) proteins expressed in *Escherichia coli*. Antiphosphothreonine and antiphosphotyrosine immunoblots showing patterns of auto- and transphosphorylation. Not all receptor kinases were capable of transphosphorylating proteins following induction in *E. coli*. Auto- and transphosphorylation activity of recombinant and *E. coli* proteins was measured after receptor kinases had been expressed for 16 h. Each sample shows total crude proteins, including FLAG-tagged receptor kinase, extracted from *E. coli* and subjected to 12% sodium dodecyl sulfate polyacrylamide gel electrophoresis (SDS-PAGE) before transfer to polyvinylidene difluoride (PVDF) membranes. Membranes were immunoblotted (IB) with the antibodies indicated. The asterisks (*) indicate which leucine-rich repeat receptor-like kinases (LRR-RLKs) shown autophosphorylation or auto-and transphosphorylation.

**Figure 2:**
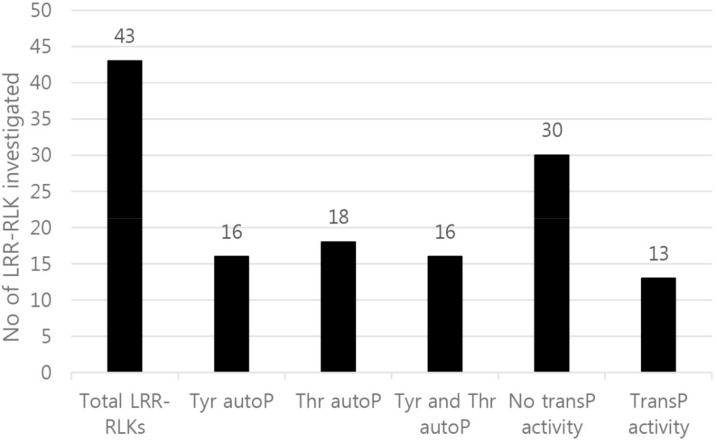
Analysis of auto- and transphosphorylation activity of 43 receptor kinases. All kinases were expressed as recombinant fusion proteins with an N-terminal Flag tag. Not all LRR-RLK proteins had cytoplasmic domains (CDs) capable of autophosphorylation activity in *E. coli*; only LRR-RLKs with autophosphorylation activity showed transphosphorylation kinase activity. P: phosphorylation.

**Figure 3:**
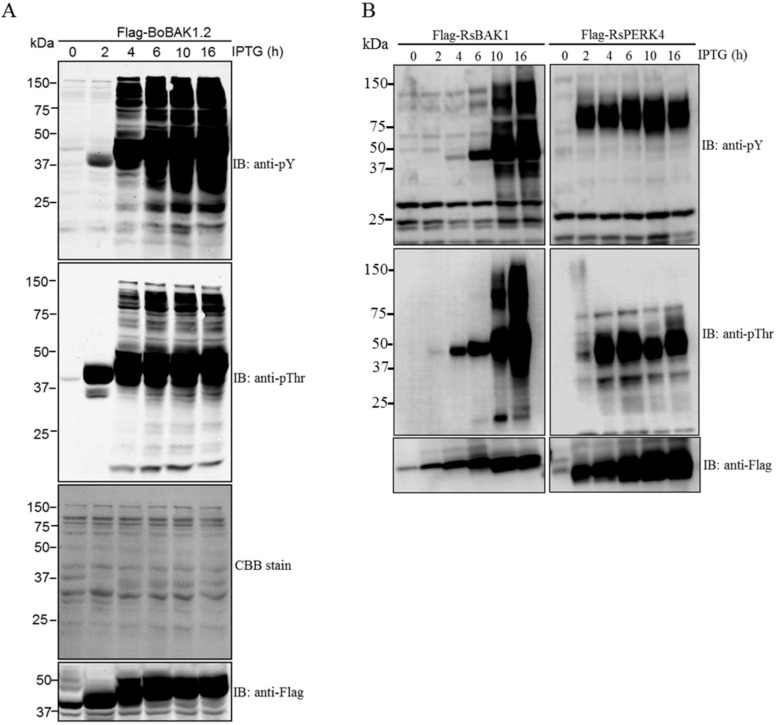
Time courses of FLAG-tagged *B. olearacea* BRI1-Associated Receptor Kinase (FLAG-BoBAK1), FLAG-tagged *R. sativus* BAK1 (FLAG-RsBAK1), and FLAG-tagged *R. sativus* Proline-rich receptor-like protein kinase (FLAG-RsPERK4) production in *E. coli*. (**A**) Analysis of FLAG-BoBAK1 kinase phosphoprotein production at different times after isopropyl β-d-1-thiogalactopyranoside (IPTG) induction. Immunoblots were probed with antibodies as indicated; (**B**) Analysis of FLAG-RsBAK1 and RsPERK4 kinase auto- and transphosphorylation kinase activity. Immunoblots show kinase activity in samples collected at the stated times after protein induction. CBB: Coomassie Brilliant Blue; pY: anti-phosphotyrosine; pThr: anti-phosphothreonine.

**Figure 4:**
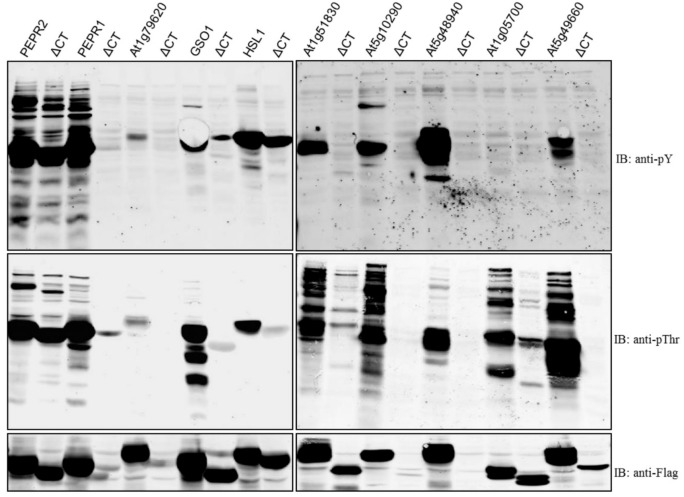
Comparison of wild-type receptor and C-terminal region-deletion kinases (ΔCT) with recombinant proteins of *A. thaliana*. Ten *Arabidopsis* LRR-RLKs were selected based on the length of the amino acid sequence of their CT regions and cloned into the protein expression vector pFlag-Mac. Immunoblots of recombinant full-length and cytoplasmic domain-deletion proteins were probed with pY or pThr antibodies.

**Figure 5:**
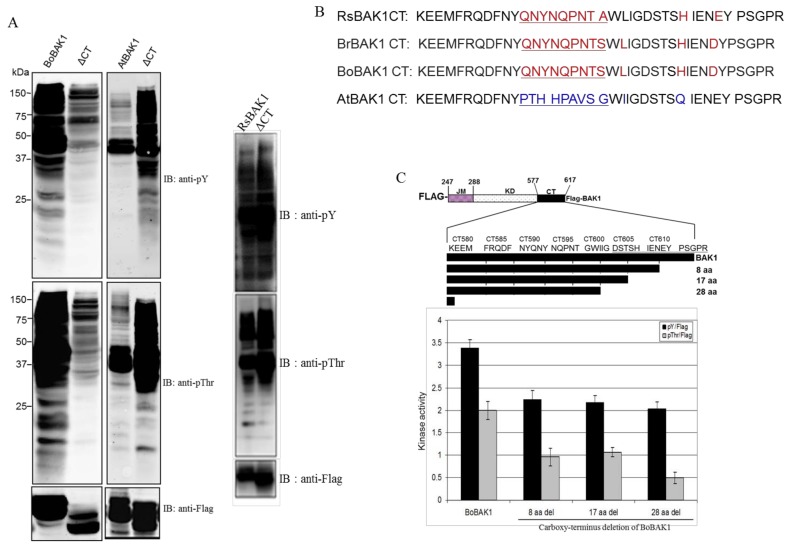
The CT region of BoBAK1 is a positive regulator and AtBAK1 is a negative regulator of recombinant BAK1 transphosphorylation activity. (**A**) Kinase activity of wild-type and CT region-deletion BAK1 from *B. oleracea*, *A. thaliana*, and *R. sativus*. Immunoblots were probed with pY or pThr, and anti-FLAG tag antibodies; (**B**) Alignment of amino acid sequences of BAK1 CT regions from four plant species (*R. sativus*, *Brassica rapa*, *B. oleracea*, and *A. thaliana*); the sequences from the first three species are very similar and differ greatly from AtBAK1; (**C**) Schematic representation of the Flag-BoBAK1 truncated CD used in these experiments. Residue numbers at the domain interfaces are shown. Phospho-amino acid signals are normalized against the amount of recombinant protein, determined by cross-reaction with anti-FLAG antibodies. JM: juxtamembrane domain; KD: kinase domain.

As an initial experimental approach to characterizing the biochemical role of the CD of the LRR-RLK CT region, we used recombinant LRR-RLK proteins from *B. oleracea*, *A. thaliana*, and *R. sativus* with intact CDs and a set of deletion mutants lacking the CT domain. All recombinant proteins contained the KD and had a FLAG-epitope tag at the terminus (Figure 1, Figure 2, Figure 3, Figure 4 and Figure 5).

In the beginning research stage, we would like to monitor auto- and transphosphorylation in the LRR-RLKs protein in *E. coli*. Therefore, we used total crude extract for Western blot analysis instead of purified proteins. In terms of purity after Flag immunopurification, probably, the purity is above 90%.

We selected RD type LRR-RLKs, which is conserved amino acids, arginine(R)-aspartic acid(d) in subdomain of kinase region in LRR-RLKs and 43 genes cloned well in this study. Eventually, we cloned 43 full-length complementary DNA (cDNA) sequences from *B. oleracea* to analyze patterns of auto- and transphosphorylation activity and kinase activities in vitro. Not all LRR-RLK CDs possessed autophosphorylation activity, and of the 18 that did, only 13 also showed transphosphorylation activity in *Escherichia coli* (Figure 1 and Figure 2; Table S1). As shown at Figure 1, protein expression level of some LRR-RLKs is too low, it is that possibly phosphoThr and phosphoTyr antibodies couldn't detect as well although two phosphoThr and phosphoTyr antibodies are very sensitive. Autophosphorylation of BoLRR21 kinase occurred only at tyrosine residue(s), indicating that this receptor kinase did not autophosphorylate at threonine residue(s); however, immunoblots of other LRR-RLKs showed autophosphorylation at Thr, and Tyr residue(s), indicating that some proteins had multi-specific kinase activity. BoLRR5 (BoBAK1) and *R. sativus* BAK1 (RsBAK1) showed strong patterns of auto- and transphosphorylation and kinase activity following the induction of recombinant protein expression in *E. coli* by isopropyl β-d-1-thiogalactopyranoside (IPTG) (Figure 1 and Figure 3). BoBAK1 showed autophosphorylation at threonine and tyrosine residue(s) 2 h after the addition of IPTG, and transphosphorylation kinase activity was significantly increased 4 h after the induction of recombinant protein (Figure 3A). This was unexpected in comparison with the kinase activity of other LRR-RLKs, including RsBAK1 and *R. sativus* proline-rich receptor-like protein kinase (RsPERK4), although RsBAK1 had strong auto- and trans-phosphorylation at threonine and tyrosine residues (Figure 3B).

Although the functions of autophosphorylation are not well understood, autophosphorylation of receptor kinases is recognized to be an important process in plant receptor kinase signaling. Many LRR-RLKs, including BAK1, autophosphorylate tyrosine residues, as well as at many Ser/Thr residues [7]. Surprisingly, tyrosine phosphorylation in plants is now known to be more abundant than first indicated by overviews of rice and *Arabidopsis* phosphoproteomes [12,13]. The plant receptor kinase, BAK1, is a partner of BRI1, the flagellin receptor (FLS2), and PEPR1. It is a co-receptor and positive regulator of several signaling pathways, including BR and innate immunity signaling, as well as a negative regulator of programmed cell death [14]. It was shown recently that the sugar transporters Sugar transport protein 1 (STP1) and STP13 contribute to an antibacterial defense mechanism, and STP13 activity is induced in response to flg22. Moreover, STP13 participates in FLS2 complexes and is phosphorylated by BAK1. Regulation of STP13 activity by T485 phosphorylation is required to suppress bacterial proliferation, partly by limiting virulence factor delivery [15]. In addition, BAK1 interacts with Open Stomata 1 (OST1) near the plasma membrane and levels of BAK1/OST1 complexes increase in response to abscisic acid (ABA) in planta. Moreover, BAK1 and ABI1 oppositely regulate OST1 phosphorylation in vitro, and the interaction of ABI1 with BAK1 inhibits the interaction of BAK1 and OST1 [16]. Therefore, studies of the BAK1 receptor kinase in *B. oleracea*, *A. thaliana*, and *R. sativus* are extremely important for understanding its functional role in vivo.

To provide further insight into this process, ten *Arabidopsis* LRR-RLKs were selected based on the length of the CT polypeptide and cloned into the pFlag-Mac protein expression vector. Recombinant proteins containing the CD were compared with recombinant protein lacking the CT region (Figure 4). We found that GSO1, HSL1, At1g51830, At1g05700, and At5g49660 showed autophosphorylation kinase activity; by contrast, kinase activity was lost in a set of deletion mutants lacking the CT region (Figure 4 and Table 1; four clones produced poor protein expression). These results indicated that the CT region regulated LRR-RLK activity in *A. thaliana* and suggested that this region played an important role in diverse signal transduction pathways. The length of the CT polypeptide was not an indication of its ability to regulate kinase activity, although the CT region of At4g20140 (GSO1) contained nine amino acids whereas that of At1g51830 contained 34 amino acids, both recombinant proteins lost autophosphorylation kinase activity in the absence of the CT region (Figure 4 and Table 1). The peptide hormones Casparian Strip Integrity Factor 1 (CIF1) and CIF2 are expressed in the root stele and specifically bind the endodermis-expressed LRR receptor kinase Gassho1 (GSO1)/Schengen3 and its homolog GSO2 [17]. These are good candidates for further study of the molecular and biochemical mechanisms of Casparian strip diffusion barrier formation in *Arabidopsis* roots using a protein phosphorylation analysis approach.

To understand the role of the CT domain in *B. oleracea*, *A. thaliana*, and *R. sativus* LRR-RLK proteins, we used a FLAG-tagged BAK1 CD construct to produce a set of deletion mutants lacking the CT polypeptide. A comparison of these proteins showed distinct differences in auto- and transphosphorylation kinase activity between *B. oleracea* and *A. thaliana* BAK1 CDs (Figure 5A). The BoBAK1 CD showed stronger auto- and transphosphorylation at threonine and tyrosine residues than the CT-deletion protein, which suggested that the CT region was critical for the regulation of kinase activity and subsequent diverse signaling cascades, including plant growth, pathogen responses, and programmed cell death in *B. oleracea.* By contrast, AtBAK1 CD showed autophosphorylation at threonine and tyrosine residues but weak transphosphorylation activity, and this CT was a strong inhibitor of transphosphorylation activity in particular (Figure 5A).

Sequence alignment of the BAK1 CT regions from four different plant species showed that the amino acid sequences of RsBAK1, BrBAK1, and BoBAK1 resembled each other very closely but differed from the AtBAK1 CT sequence between amino acids 588 and 596, as well as at the amino acids I598, Q605 and E609 (Figure 5B). As a further means of identifying the regulatory region of the BoBAK1 CT domain, we constructed a serial truncation series that sequentially deleted 8, 17 and 28 amino acids from the flanking region (Figure 5C). Our results suggested that the final eight amino acids of the CT domain were responsible for activating transphosphorylation activity; in particular, it was likely that aspartic acid (D) played a critical role in regulating BoBAK1 kinase activity as, in AtBAK1, D was replaced by glutamic acid (E). As a result, in an *E. coli* expression system, kinase activity resulting in transphosphorylation of threonine residues was significantly reduced by deletion of the CT region.

**Figure 6:**
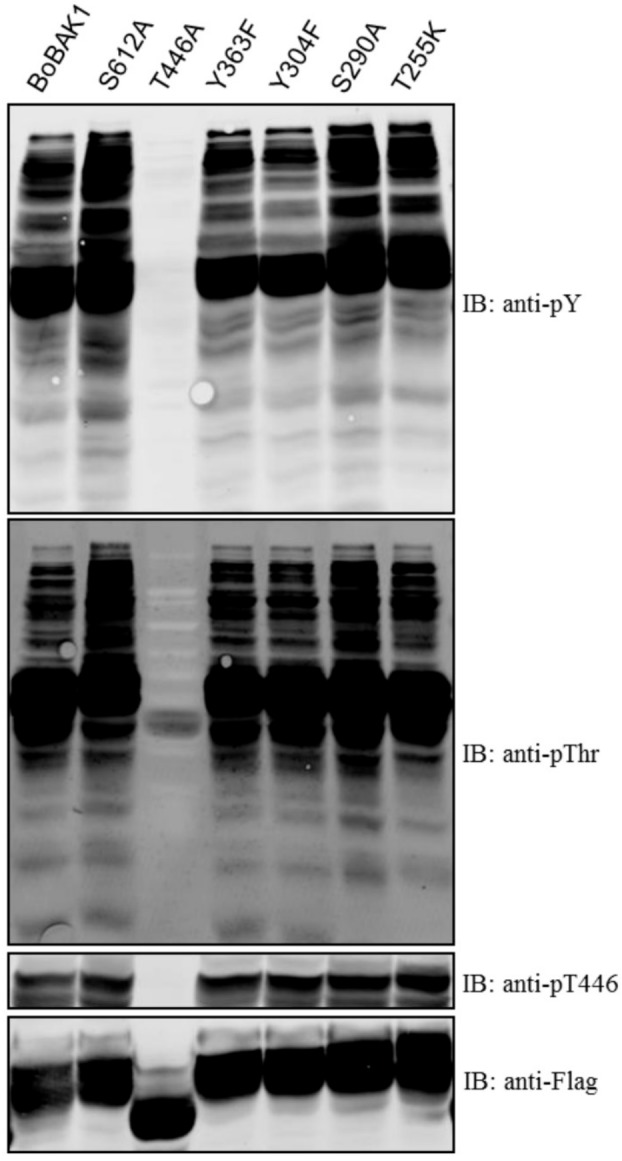
Effect on auto- and transphosphorylation of the recombinant proteins of site-directed mutagenesis in the cytoplasmic domain of FLAG-BoBAK1. Immunoblots were probed with pY or pThr antibodies. Recombinant proteins were detected by immunoblotting with anti-Flag antibodies.

Collectively, our results suggested that the CT domain was a strong activator of transphosphorylation in BoBAK1 but a strong inhibitor in AtBAK1. As reported by Wang et al. [7], the coreceptor BAK1 is activated by BRI1-dependent transphosphorylation and subsequently enhances signaling output through reciprocal BRI1 transphosphorylation. These authors identified AtBAK1 phosphorylation sites using Ion Trap or Quadrupole Time-of-Flight (Q-ToF) Liquid Chromatography/Mass Spectrometry/Mass Spectrometry (LC/MS/MS) in vitro and in vivo. The BAK1 phosphorylation sites were within the KD, including S290 in subdomain I; at T312 in subdomain II; and at three residues, T446, T449, and T455, in the activation loop of subdomains VII/VIII [18]. To understand the importance of individual BoBAK1 phosphorylation sites for kinase activity, we performed site-directed mutagenesis at specific residues by substituting serine or threonine with alanine (S612A, S290A and T446A) or by replacing tyrosine with phenylalanine (Y363F and Y304F). Notably, BoBAK1 with S290A and S612A substitutions showed higher transphosphorylation kinase activity than the wild-type protein, suggesting that kinase activity may be inhibited at both phosphorylation sites in vitro. Surprisingly, T446A-substituted BoBAK1 had no auto- or transphosphorylation, implying that the T446 residue was essential for regulating kinase activity (Figure 6).

Our previous studies of the flanking domains of BRI1 found that JMs activate kinase activity [8,11]. Our current results indicated that the CT regions of Arabidopsis LRR-RLKs, including AtBAK1, inhibited kinase activity. Overall, our results suggested that the LRR-RLK CT domain plays an important role in regulating kinase activity in vitro. CT epitope tags may affect BAK1 function in different signaling pathways since BAK1 also functions as a co-receptor with the LRR-RLKs FLS2 and Elongation Factor TU Receptor (EFR) [19,20]. Therefore, it will be interesting to determine whether the CT regions of BAK1 and other LRR-RLKs are essential for signaling pathways and our future work shall explore this in vivo.

## 3. Material and Methods

### 3.1. Isolation of Total RNA and Cloning of LRR-RLKs

Total RNA was extracted from leaf tissue of *A. thaliana*, *R. sativus* L., and *B. oleracea* var. *capitata* using TRIzol reagent (Applied Biosystems, Carlsbad, CA, USA) according to the manufacturer’s instructions [21]. Single-strand cDNA was obtained by reverse transcribing 2 μg of DNase-treated RNA using Superscript III reverse transcriptase (Applied Biosystems).

Gene sequences of BoLRR-RLKs were obtained from the *Brassica* database and compared using BLAST and TBLASTN tools with the *Arabidopsis* genome information (AGI) CDS dataset to confirm their sequence identity [14]. The selected genes were synthesized using gene-specific primers and AccuPrime Pfx DNA Polymerase (Invitrogen, Carlsbad, CA, USA) with polymerase chain reaction (PCR) conditions of 95 °C for 2 min, 35 cycles of 95 °C for 20 s, 60 °C for 40 s, and 72 °C for 2 min, and a final extension phase at 72 °C for 10 min. The amplified fragments were purified and cloned into the pENTR-D-TOPO vector (Invitrogen) and confirmed by sequence analysis. With DNA recombination using Clonase II enzyme (Invitrogen, Carlsbad, CA, USA) between entry clone and final destination protein expression, finally cloned into the pFLAG-Mac protein expression vector to enable production of recombinant proteins in *E. coli*.

### 3.2. Recombinant Protein Production and Purification

To produce LRR-RLK recombinant proteins, *E. coli* BL21 (DE3) (Novagen, Temecula, CA, USA) cells were transformed with vectors containing the genes of interest. *E. coli* cells were grown in Luria-Bertani (LB) medium, and expression of receptor kinases was induced with 0.3 mM Isopropyl β-d-1-thiogalactopyranoside (IPTG) (Sigma-Aldrich, St. Louis, MO, USA) when the optical density (OD_600_) of the cell culture reached 0.6. For time series analysis of activity, *E. coli* cells were incubated at room temperature with shaking for the indicated times (up to 16 h) following IPTG induction. Cells were harvested by centrifugation and resuspended in a buffer containing 50 mM 3-(N-morpholino) propanesulfonic acid (MOPS) (pH 7.5), 150 mM NaCl, and protease inhibitors before being lysed by sonication. Cell lysates were fractionated by centrifugation at 35,000× *g* into soluble and pellet fractions. The recombinant FLAG-tagged protein kinases in the soluble fractions were immunopurified on an anti-FLAG M2 affinity gel (Sigma-Aldrich, St. Louis, MO, USA). After purification, the protein solutions were dialyzed against a 1000× volume of dialysis buffer containing 20 mM MOPS (pH 7.5) and 1 mM dithiothreitol (DTT) (Sigma-Aldrich, St Louis, MO, USA), as previously described [9].

### 3.3. Electrophoresis and Immunoblotting

Recombinant protein preparations were mixed with pre-heated (95 °C) 1× sodium dodecyl sulfate polyacrylamide gel electrophoresis (SDS-PAGE) sample buffer containing 1 M urea, 0.7 M 2-mercaptoethanol, 5 mM NaF, 1 mM Na_2_MoO_4_, 1 mM Na_3_VO_4_, 1 mM aminoethylbenzenesulfonyl fluoride, and 2 mM EDTA. Protein concentrations were determined by the dye-binding assay (Bio-Rad, Hercules, CA, USA) using bovine serum albumin as a protein standard. Proteins were separated on 12% polyacrylamide (0.1% SDS) gels and transferred to polyvinylidene difluoride (PVDF) fluorescence-specific membranes (Millipore, Bedford, MA, USA). Membranes were blocked in a 2% (*v*/*v*) fish gelatin solution in phosphate-buffered saline (PBS; 5 mM NaH_2_PO_4_, 150 mM NaCl, pH 7.4) before being incubated with primary antibodies, which were diluted as specified in PBS containing 0.1% (*v*/*v*) Tween-20 (PBST). Purified recombinant proteins or proteins in the soluble fraction were analyzed using SDS-PAGE and immunoblotting with anti-FLAG antibodies (1:5000 dilution), antiphosphothreonine antibodies (1:500 dilution), and antiphosphotyrosine antibodies (1:500 dilution) to monitor the overall pattern of phosphorylation and level of recombinant protein expression in *E. coli*. Immunoblots were scanned using an Odyssey C-Digit scanner (LI-COR Bioscience, Lincoln, NE, USA) for visualization.

### 3.4. Phylogenetic Tree Construction

Neighbor-joining (NJ) phylogenetic tree was constructed by multiple alignment analysis with 1000 iterations using the ClustalX 2.0 program [22] and the tree was generated using the Molecular Evolutionary Genetics Analysis (MEGA) 6 software [23].

## Figures and Tables

**Table 1:** Summary of wild-type leucine-rich repeat receptor-like kinases (LRR-RLK) and cytoplasmic domain-deletion mutants from *Arabidopsis thaliana*.

Gene Locus	Symbol	Total Amino Acids	Amino Acids of CT	CD autoP	ΔCT autoP
At1g17750	PEPR2	1088	8	pThr/pY	pThr/pY
At1g73080	PEPR1	1123	8	pThr/pY	poor expression
At1g79620		971	59	pThr/pY	poor expression
At4g20140	GSO1	1249	9	pThr/pY	ND
At1g28440	HSL1	996	34	pThr/pY	pY only
At1g51830		882	34	pThr/pY	ND
At5g10290		613	44	pThr/pY	poor expression
At5g48940		1135	69	pThr/pY	poor expression
At1g05700		852	7	pThr	Reduced pThr
At5g49660	CEPR1	966	32	pThr/pY	ND

PEPR1: PEP1 RECEPTOR 1; PEPR2: PEP2 RECEPTOR 2; GSO1: GASSHO 1; HSL1: HAESA-LIKE 1; CEPR1: CEP RECEPTOR 1; autoP: autophosphorylation; pThr/pY: phosphorylation of Threonine and Tyrosine residue(s); ND: Not detected; ΔCT: Deletion of Carboxy-Terminus.

## Supplementary Materials

Supplementary Materials are available online.
