# Supplementary material for: Biochemical Analysis of the Role of Leucine-Rich Repeat Receptor-Like Kinases and the Carboxy-Terminus of Receptor Kinases in Regulating Kinase Activity in Arabidopsis thaliana and Brassica oleracea
Source: Molecules. 2018 Jan 22;23(1):236. doi: 10.3390/molecules23010236 (PMC6017770; doi:10.3390/molecules23010236)
Supplement: Supplementary file 1 [file molecules-23-00236-s001.pdf]

Biochemical Analysis of the Role of Leucine-Rich Repeat Receptor-Like Kinases and the Carboxy-Terminus of Receptor Kinases in Regulating Kinase Activity in *Arabidopsis thaliana* and *Brassica oleracea*

Eun-Seok Oh [1,†], Yeon Lee [1,†], Won Byoung Chae [2,†], Jana Jeevan Rameneni [3], Yong-Soon Park [1], Yong Pyo Lim [3] and Man-Ho Oh [1,*]

[1] Department of Biological Sciences, College of Biological Sciences and Biotechnology, Chungnam National University, Daejeon 34134, Korea; oes0318@naver.com (E.-S.O.); yeonlee@cnu.ac.kr (Y.L.); yspark2005@gmail.com (Y.-S.P.)

[2] Vegetable Research Division, National Institute of Horticultural and Herbal Science, RDA, Wanju, Korea; chaeddang@korea.kr

[3] Department of Horticulture, College of Agriculture and Life Science, Chungnam National University, Daejeon, Korea; saijeevan7@gmail.com (J.J.R.); yplim@cnu.ac.kr (Y.P.L.)

* Correspondence: manhooh@cnu.ac.kr; Tel.: +82-42-821-5497

† These authors contributed equally to this work.

Table S1. Summary of 43 LRR-RLKs cloned and investigated in *Brassica oleracea*.

Given name	Locus of *B. oleracea*	Locus of *A. thaliana*	Symbol	Full length CDS (bp)
BoLRR5	Bol033816	At4g33430	BAK1	1644
BoLRR10	Bol016985	At3g49670	BAM2	2976
BoLRR11	Bol021078	At1g08590	CLAVATA1	3060
BoLRR13	Bol024340	At4g20270	BAM3-2	2535
BoLRR15	Bol026354	At1g72300-1		3264
BoLRR17	Bol028337	At4g20270	BAM3-3	2904
BoLRR20	Bol031960	At1g35710		2751
BoLRR21	Bol032005	At1g34110		3297
BoLRR22	Bol032343	At5g48940	RCH1	3384
BoLRR24	Bol035223	At1g55610	BRL1	2280
BoLRR25	Bol036776	At1g09970-3		2907
BoLRR26	Bol040029	At1g73080	PEPR1	2646
BoLRR27	Bol040099	At1g34210	SERK2	1866
BoLRR30	Bol025935	AT5G20480	EFR1-1	2685
BoLRR31	Bol025932	AT5G20480	EFR1-2	2685
BoLRR32	Bol032113	AT5G46330	FLS2	3531
BoLRR33	Bol033102	AT5G48380	BIR1	1878
BoLRR35	Bol015128	AT2G02220	PSKR1-2	2817
BoLRR36	Bol020960	At3g46350		2212
BoLRR38	Bol003589	At1g72180-1		2922
BoLRR39	Bol040075	At1g72180-2		2925
BoLRR40	Bol040522	At1g79620		2373
BoLRR41	Bol001136	At2g01950	BRL2	3273
BoLRR44	Bol008219	At2g31880	SOBIR1	1932
BoLRR46	Bol005575	At2g37050-2		2517
BoLRR48	Bol028329	At4g20140	GSO1	3617
BoLRR53	Bol040377	At3g13380	BRL3	2160

Table S1. *Cont.*

BoLRR54	Bol017009	At5g61480	TDR	2625
BoLRR55	Bol004350	At5g65700	PXL2	2984
BoLRR56	Bol012386	At5g44700-1	GSO2-1	3671
BoLRR57	Bol006354	At5g44700-2	GSO2-2	3789
BoLRR58	Bol043860	At5g07180	ERL1	2477
BoLRR59	Bol042730	At3g25560	NIK2	1733
BoLRR60	Bol025602	At4g08850		3077
BoLRR61	Bol019648	At4g28650	PXL2	3035
BoLRR63	Bol024650	At5g10290		1812
BoLRR65	Bol012709	At5g25930-2		2732
BoLRR67	Bol002765	At5g49660		2783
BoLRR70	Bol003055	At5g63710		1653
BoLRR71	Bol040608	At5g63930		2912
BoLRR72	Bol009214	At5g65240		1817
BoLRR73	Bol041836	At1g28440-1	HSL1	2987
BoLRR74	Bol015744	At1g28440-2	HSL1-2	2629

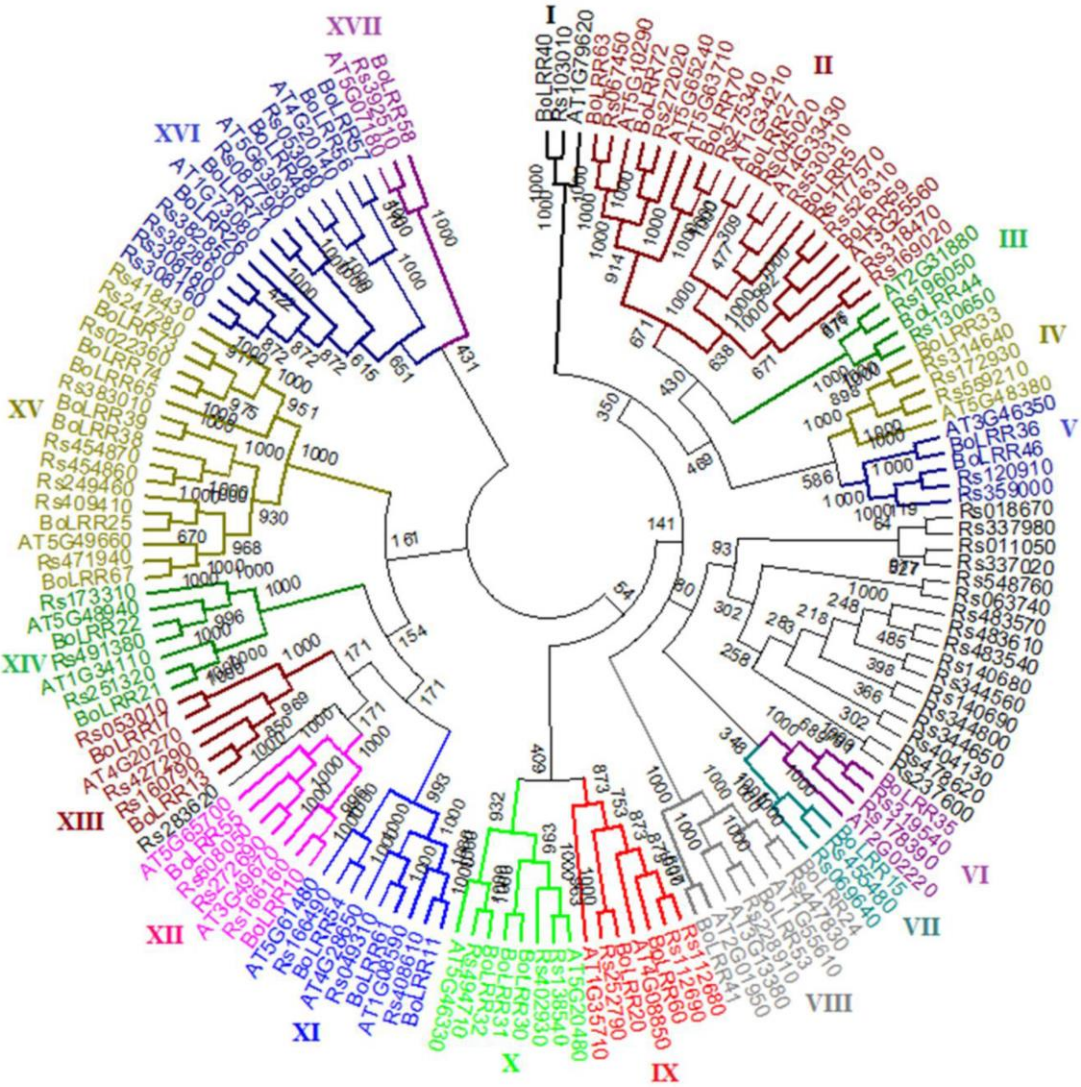

Figure S1. Comparative phylogeny analysis of LRR-RLK genes of *A. thaliana*, *B. oleracea*, and *R. sativus*
